# Supplementary material for: Major antigenic site B of human influenza H3N2 viruses has an evolving local fitness landscape
Source: Nat Commun. 2020 Mar 6;11:1233. doi: 10.1038/s41467-020-15102-5 (PMC7060233; doi:10.1038/s41467-020-15102-5)
Supplement: Supplementary file 1 — Supplementary Information [file 41467_2020_15102_MOESM1_ESM.pdf]

Supplementary information

Major antigenic site B of human influenza H3N2 viruses has an evolving local fitness landscape

Nicholas C. Wu[1], Jakub Otwinowski[2], Andrew J. Thompson[3], Corwin M. Nycholat[3], Armita Nourmohammad[2,4], Ian A. Wilson[1,5,§]

[1] Department of Integrative Structural and Computational Biology, The Scripps Research Institute, La Jolla, CA 92037, USA

[2] Max Planck Institute for Dynamics and Self-Organization, am Faßberg 17, 37077 Göttingen, Germany

[3] Department of Molecular Medicine, The Scripps Research Institute, 10550 North Torrey Pines Road, La Jolla, CA 92037, USA

[4] Department of Physics, University of Washington, 3910 15th Ave Northeast, Seattle, WA 98195, USA

[5] The Skaggs Institute for Chemical Biology, The Scripps Research Institute, La Jolla, CA, 92037, USA

[§] Correspondence: wilson@scripps.edu (I.A.W.)

Supplementary Table 1. X-ray data collection and refinement statistics.

**Data collection**	A/Hong Kong/1/1968 + 6′SLNLN
Beamline	APS 23ID-B
Wavelength (Å)	1.0332
Space group	C2
Unit cell parameters (Å and °)	a=210.4, b=132.2, c=72.5, β=97.9
Resolution (Å)	50.00-2.25 (2.34-2.25)[a]
Unique Reflections	93,371 (10,355)[a]
Redundancy	6.5 (6.0)[a]
Completeness (%)	100.0 (100.0)[a]
$<I/\sigma_I>$	16.3 (1.5)[a]
R_{sym}[b]	0.10 (0.90)[a]
R_{pim}[b]	0.04 (0.40)[a]
$CC_{1/2}$[c]	0.99 (0.61)[a]
Z_a[d]	3
**Refinement statistics**	
Resolution (Å)	44.11-2.25
Reflections (work)	88,692
Reflections (test)	4,625
R_{cryst}(%)[e] / R_{free}(%)[f]	15.7 / 20.7
No. of atoms	
Protein	11,520
Water	919
Glycan	371
6′SLNLN	188
Average *B*-value (Å^2)	
Protein	47
Water	47
Glycan	94
6′SLNLN	82
Wilson *B*-value (Å^2)	31
**RMSD from ideal geometry**	
Bond length (Å)	0.008
Bond angle (°)	0.97
**Ramachandran statistics (%)**[g]	
Favored	96.5
Outliers	0.2
**PDB code**	6TZB

[a] Numbers in parentheses refer to the highest resolution shell.

[b] $R_{sym} = \Sigma_{hkl} \Sigma_i | I_{hkl,i} - <I_{hkl}> | / \Sigma_{hkl} \Sigma_i I_{hkl,i}$ and $R_{pim} = \Sigma_{hkl} (1/(n-1))^{1/2} \Sigma_i | I_{hkl,i} - <I_{hkl}> | / \Sigma_{hkl} \Sigma_i I_{hkl,i}$, where $I_{hkl,i}$ is the scaled intensity of the ith measurement of reflection h, k, l, $<I_{hkl}>$ is the average intensity for that reflection, and *n* is the redundancy.

[c] $CC_{1/2}$ = Pearson correlation coefficient between two random half datasets.

[d] Z_a is the number of HA protomers per crystallographic asymmetric unit.

[e] $R_{cryst} = \Sigma_{hkl} | F_o - F_c | / \Sigma_{hkl} | F_o | \times 100$, where F_o and F_c are the observed and calculated structure factors, respectively.

[f] R_{free} was calculated as for R_{cryst}, but on a test set comprising 5% of the data excluded from refinement.

[g] Calculated with MolProbity[1].

Supplementary Table 2. Primers for deep mutational scanning.

Strain	PCR reaction	Primer type	Sequence
HK68	Mutant library insert	Forward	a. 5'-CGT ACG TCT CAT GAC CCA WTC AAA WTH TAC ATA TCC AGT GCT GAA CGT GAC-3'<br>b. 5'-CGT ACG TCT CAT GAC CCA WTC AGR ATH TAC ATA TCC AGT GCT GAA CGT GAC-3'<br>c. 5'-CGT ACG TCT CAT GAC CRA ATC AAA WTH TAC ATA TCC AGT GCT GAA CGT GAC-3'<br>d. 5'-CGT ACG TCT CAT GAC CRA ATC AGR ATH TAC ATA TCC AGT GCT GAA CGT GAC-3'
		Reverse	a. 5'-CGT ACG TCT CAG CTT GTR CAT ACA GAR AGG TTT GWT CTT GGT TCG TGC TCG GGT GGT GAA-3'<br>b. 5'-CGT ACG TCT CAG CTT GTR CAT ACA GAT TGG TTT GWT CTT GGT TCG TGC TCG GGT GGT GAA-3'
	Mutant library vector	Forward	5'-CGT ACG TCT CAG TCA ACC AGT TCA GTC TAC TGA AAA-3'
		Reverse	5'-CGT ACG TCT CAA AGC ATC AGG GAG AGT CAC AGT CTC-3'
	Sequencing preparation	Forward	5'-CAC TCT TTC CCT ACA CGA CGC TCT TCC GAT CTT TCA GTA GAC TGA ACT GGT TGA-3'
		Reverse	5'-GAC TGG AGT TCA GAC GTG TGC TCT TCC GAT CTG AGA CTG TGA CTC TCC CTG ATG-3'
Bk79	Mutant library insert	Forward	a. 5'-CGT ACG TCT CAT GTA CCA WTC AAA WTH TAA ATA TCC AGT GCT GAA CGT GAC-3'<br>b. 5'-CGT ACG TCT CAT GTA CCA WTC AGR ATH TAA ATA TCC AGT GCT GAA CGT GAC-3'<br>c. 5'-CGT ACG TCT CAT GTA CRA ATC AAA WTH TAA ATA TCC AGT GCT GAA CGT GAC-3'<br>d. 5'-CGT ACG TCT CAT GTA CRA ATC AGR ATH TAA ATA TCC AGT GCT GAA CGT GAC-3'
		Reverse	a. 5'-CGT ACG TCT CAG CTC GTR CAT ATA GAR AGG TTT GWT CTT TGT CCG TGC TCG GGT GGT GAA-3'<br>b. 5'-CGT ACG TCT CAG CTC GTR CAT ATA GAT TGG TTT GWT CTT TGT CCG TGC TCG GGT GGT GAA-3'
	Mutant library vector	Forward	5'-CGT ACG TCT CAT ACA ACC AAT TCA GTC TAC TGA AGA-3'
		Reverse	5'-CGT ACG TCT CAG AGC ATC AGG GAG AGT CAC AGT CTC-3'
	Sequencing preparation	Forward	5'-CAC TCT TTC CCT ACA CGA CGC TCT TCC GAT CTT TCA GTA GAC TGA ATT GGT TGT-3'
		Reverse	5'-GAC TGG AGT TCA GAC GTG TGC TCT TCC GAT CTG AGA CTG TGA CTC TCC CTG ATG-3'
Bei89	Mutant library insert	Forward	a. 5'-CGT ACG TCT CAT TGC ACC AWT CAA AWT HTA AAT ATC CAG CGC TGA ACG TGA-3'<br>b. 5'-CGT ACG TCT CAT TGC ACC AWT CAG RAT HTA AAT ATC CAG CGC TGA ACG TGA-3'<br>c. 5'-CGT ACG TCT CAT TGC ACR AAT CAA AWT HTA AAT ATC CAG CGC TGA ACG TGA-3'<br>d. 5'-CGT ACG TCT CAT TGC ACR AAT CAG RAT HTA AAT ATC CAG CGC TGA ACG TGA-3'
		Reverse	a. 5'-CGT ACG TCT CAG CTC GTR CAT ATA GAR AGG TTT GWT CTC TGT CCG TGC TCG GGT GGT GAA-3'<br>b. 5'-CGT ACG TCT CAG CTC GTR CAT ATA GAT TGG TTT GWT CTC TGT CCG TGC TCG GGT GGT GAA-3'
	Mutant library vector	Forward	5'-CGT ACG TCT CAG CAA CCA ATT CAA TCT ACT AAA GAA-3'
		Reverse	5'-CGT ACG TCT CAG AGC ATC AGG GAG AGT CAC AGT CTC-3'
	Sequencing preparation	Forward	5'-CAC TCT TTC CCT ACA CGA CGC TCT TCC GAT CTT TTA GTA GAT TGA ATT GGT TGC-3'
		Reverse	5'-GAC TGG AGT TCA GAC GTG TGC TCT TCC GAT CTG AGA CTG TGA CTC TCC CTG ATG-3'
Mos99	Mutant library insert	Forward	a. 5'-CGT ACG TCT CAT TGC ACC AWT TAA AWT HTA GAT ATC CAG CAC TGA ACG TGA-3'<br>b. 5'-CGT ACG TCT CAT TGC ACC AWT TAG RAT HTA GAT ATC CAG CAC TGA ACG TGA-3'<br>c. 5'-CGT ACG TCT CAT TGC ACR AAT TAA AWT HTA GAT ATC CAG CAC TGA ACG TGA-3'<br>d. 5'-CGT ACG TCT CAT TGC ACR AAT TAG RAT HTA GAT ATC CAG CAC TGA ACG TGA-3'
		Reverse	a. 5'-CGT ACG TCT CAG CTT GTR CAT ATA GAR AGG TTT GWT CAC TGT CCG TAC TCG GGT GGT GAA-3'<br>b. 5'-CGT ACG TCT CAG CTT GTR CAT ATA GAT TGG TTT GWT CAC TGT CCG TAC TCG GGT GGT GAA-3'
	Mutant library vector	Forward	5'-CGT ACG TCT CAG CAA CCA ATT CAA TCT ACT AAA GAA-3'
		Reverse	5'-CGT ACG TCT CAA AGC ATC AGG GAG AGT CAC AGT CTC-3'
	Sequencing preparation	Forward	5'-CAC TCT TTC CCT ACA CGA CGC TCT TCC GAT CTT TTA GTA GAT TGA ATT GGT TGC-3'
		Reverse	5'-GAC TGG AGT TCA GAC GTG TGC TCT TCC GAT CTG AGA CTG TGA CTC TCC CTG ATG-3'
Bris07	Mutant library insert	Forward	a. 5'-CGT ACG TCT CAT GAC CCA WTT AAA WTH TAA ATA CCC AGC ATT GAA CGT GAC-3'<br>b. 5'-CGT ACG TCT CAT GAC CCA WTT AGR ATH TAA ATA CCC AGC ATT GAA CGT GAC-3'<br>c. 5'-CGT ACG TCT CAT GAC CRA ATT AAA WTH TAA ATA CCC AGC ATT GAA CGT GAC-3'<br>d. 5'-CGT ACG TCT CAT GAC CRA ATT AGR ATH TAA ATA CCC AGC ATT GAA CGT GAC-3'
		Reverse	a. 5'-CGT ACG TCT CAG CTT GTR CAT ACA GAR AGA TTT GWT CAT TGT CCG TAC CCG GGT GGT GAA-3'<br>b. 5'-CGT ACG TCT CAG CTT GTR CAT ACA GAT TGA TTT GWT CAT TGT CCG TAC CCG GGT GGT GAA-3'
	Mutant library vector	Forward	5'-CGT ACG TCT CAG TCA ACC AAT TCA ATC TAC TAA AGA-3'
		Reverse	5'-CGT ACG TCT CAA AGC ATC AGG AAG AAT CAC AGT CTC-3'
	Sequencing preparation	Forward	5'-CAC TCT TTC CCT ACA CGA CGC TCT TCC GAT CTT TTA GTA GAT TGA ATT GGT TGA-3'
		Reverse	5'-GAC TGG AGT TCA GAC GTG TGC TCT TCC GAT CTG AGA CTG TGA TTC TTC CTG ATG-3'
NDako16	Mutant library insert	Forward	a. 5'-CGT ACG TCT CAT GAC CCA WTT AAA WTH TAA ATA TCC AGC ATT AAA TGT GAC-3'<br>b. 5'-CGT ACG TCT CAT GAC CCA WTT AGR ATH TAA ATA TCC AGC ATT AAA TGT GAC-3'<br>c. 5'-CGT ACG TCT CAT GAC CRA ATT AAA WTH TAA ATA TCC AGC ATT AAA TGT GAC-3'<br>d. 5'-CGT ACG TCT CAT GAC CRA ATT AGR ATH TAA ATA TCC AGC ATT AAA TGT GAC-3'
		Reverse	a. 5'-CGT ACG TCT CAG GTT GTR CAT ACA GAR AGA TTT GWT CCT TGT CCG TAC CCG GGT GGT GAA-3'<br>b. 5'-CGT ACG TCT CAG GTT GTR CAT ACA GAT TGA TTT GWT CCT TGT CCG TAC CCG GGT GGT GAA-3'
	Mutant library vector	Forward	5'-CGT ACG TCT CAG TCA ACC AAT TTA ATC TAC TAA AGA-3'
		Reverse	5'-CGT ACG TCT CAA ACC ATC AGG AAG AAT CAC AGT ATC-3'
	Sequencing preparation	Forward	5'-CAC TCT TTC CCT ACA CGA CGC TCT TCC GAT CTT TTA GTA GAT TAA ATT GGT TGA-3'
		Reverse	5'-GAC TGG AGT TCA GAC GTG TGC TCT TCC GAT CTG ATA CTG TGA TTC TTC CTG ATG-3'

Supplementary Table 3. Human H3N2 HA sequences.

Year	Number of sequences
1968	100
1969	7
1970	5
1971	10
1972	21
1973	9
1974	10
1975	8
1976	18
1977	14
1978	6
1979	3
1980	9
1981	3
1982	12
1983	6
1984	3
1985	13
1986	9
1987	5
1988	14
1989	22
1990	11
1991	37
1992	37
1993	95
1994	71
1995	73
1996	91
1997	66
1998	79
1999	176
2000	182
2001	86
2002	240
2003	489
2004	371
2005	389
2006	184
2007	597
2008	588
2009	1,079
2010	1,179
2011	1,802
2012	2,736
2013	2,284
2014	4,194
2015	5,253
2016	5,317
2017	11,663
2018	5,542

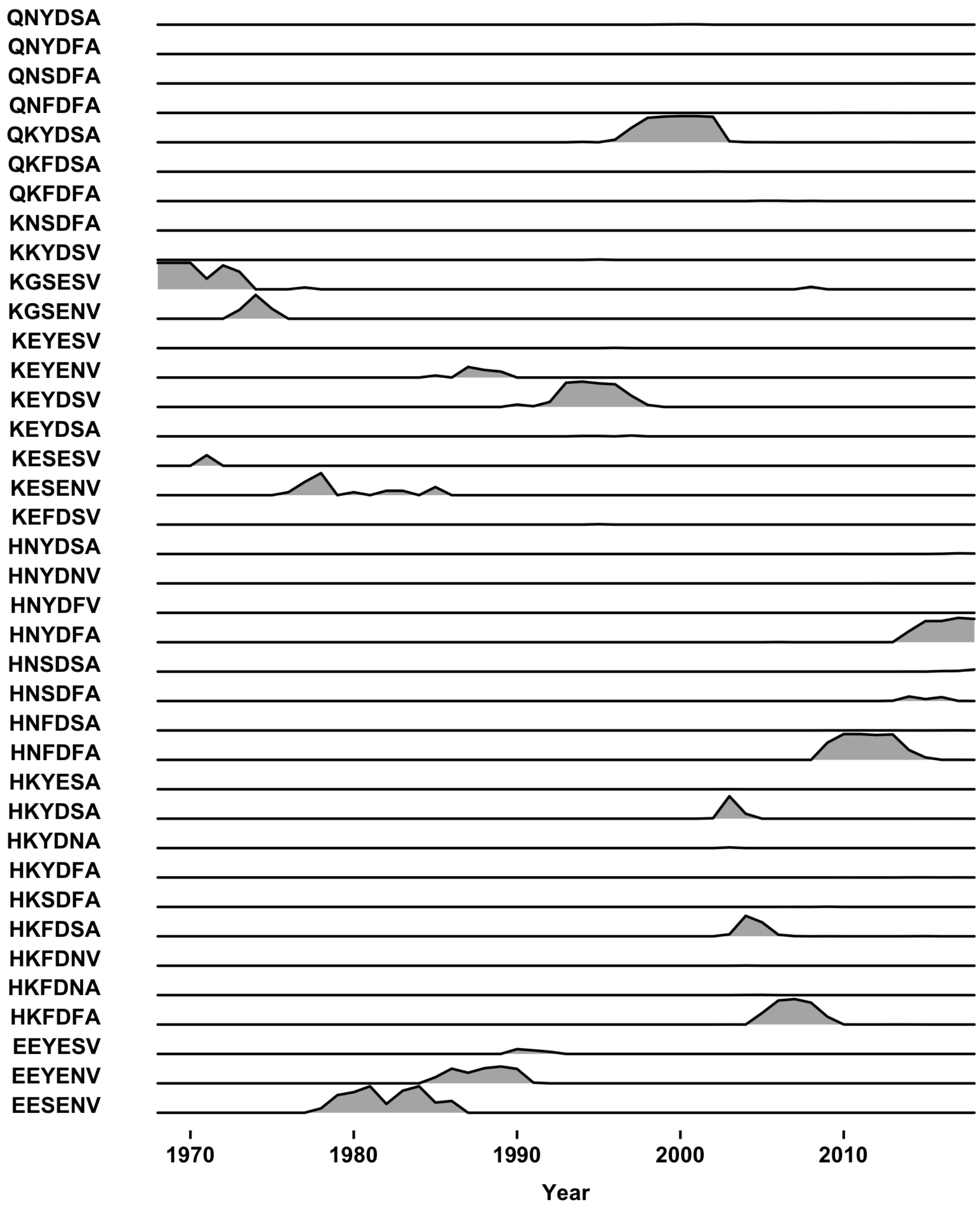

Supplementary Figure 1. Frequency of naturally occurring haplotypes in circulating human H3N2 strains. A total of 38 out of 576 haplotypes of interest have been observed in naturally circulating human H3N2 strains. The sequences of these 38 haplotypes are shown on the y-axis. Their occurrence frequencies from 1968 to 2018 are plotted. The density curve would be almost touching the next higher baseline for occurrence frequency of 100%. Of note, some haplotypes only exist at a very low frequency and therefore may look like a complete flat line in the frequency plot.

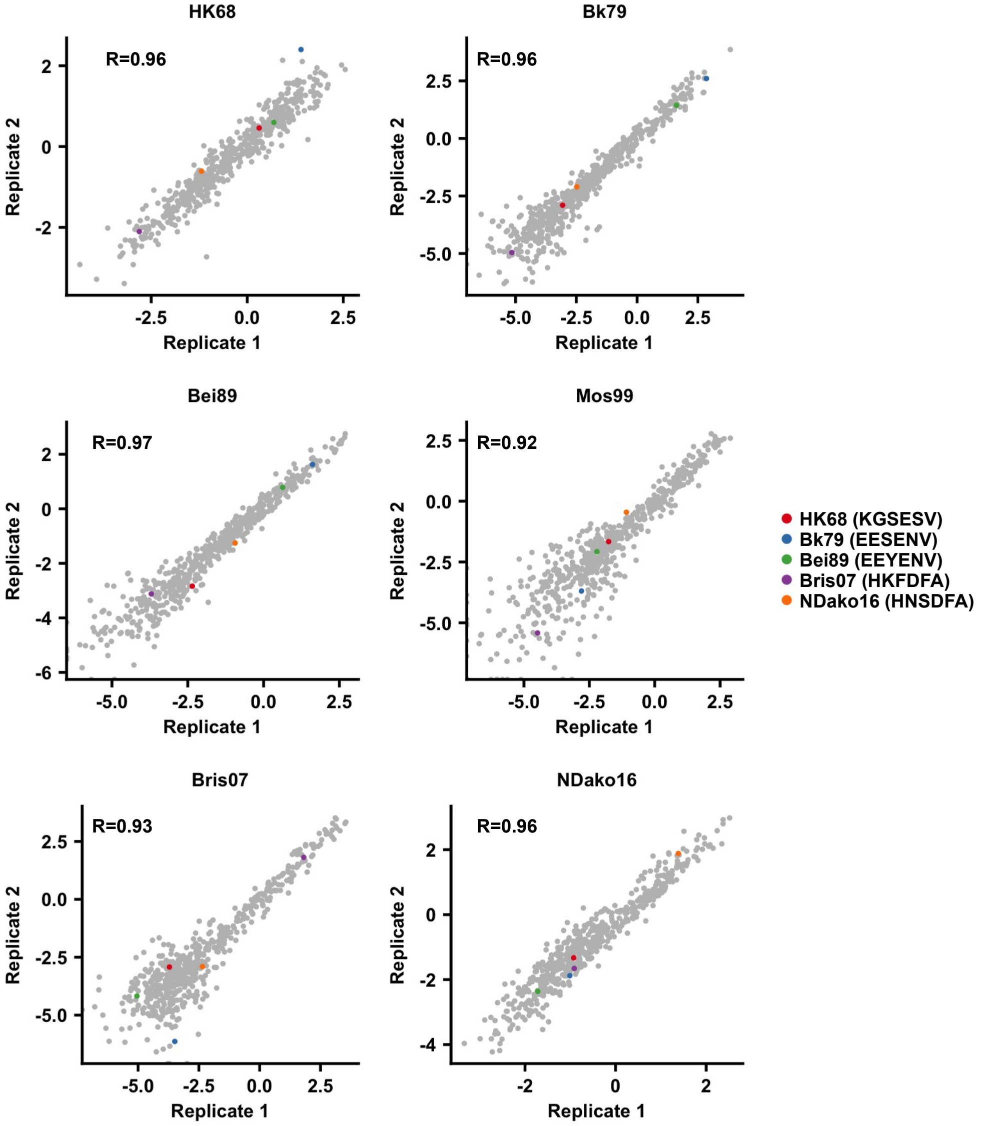

Supplementary Figure 2. Correlation between replicates. Correlations of fitness for individual variants between replicate are shown as scatterplots. Each data point within a scatterplot represents a unique variant. The Pearson correlation (R) between replicates is indicated. Data points that correspond to the WT sequences of HK68, Bk79, Bei89, Bris07, and NDako16 are colored as indicated. Of note, the WT sequence of Mos99 contains a naturally rare variant T196. Therefore, the WT sequence of Mos99 was not included in our deep mutational scanning experiment.

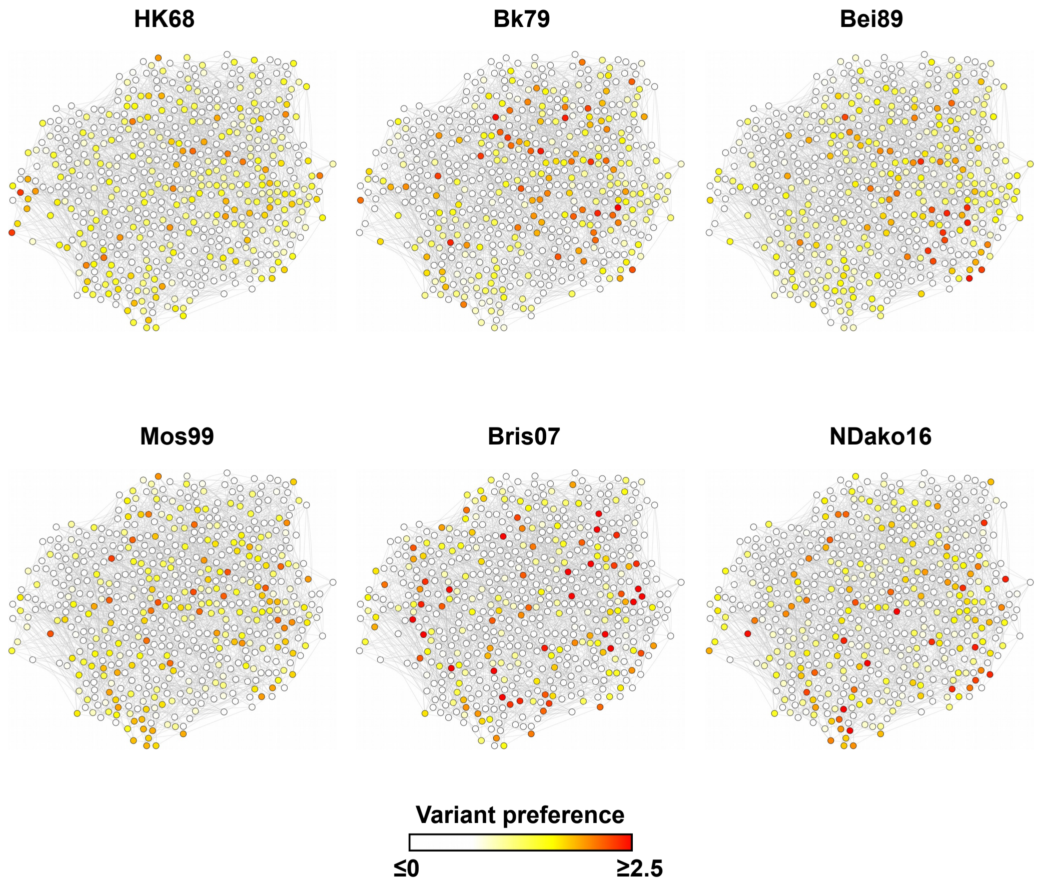

Supplementary Figure 3. Visualization of local fitness landscape using network diagram. The network diagram represents the variant preference (a proxy for replication fitness) of each of the 576 variants. Each variant is represented by a node and two nodes are connected by an edge if they are one nucleotide away from each other. The variant preference is color coded as indicated by the color bar at the bottom. For visualization purpose, variant preference of above 2.5 is shown in red, whereas variant preference of below 0 is shown in white. The topology and display arrangement of the network diagrams are the same for all six genetic backgrounds.

year of isolation	protein sequence at residues 156, 158, 159, 190, 193, 196	preference in genetic background 1	preference in genetic background 2
1968	KGSESV	-1	1
1978	EESENV	0.5	1
1988	EEYENV	1	-1

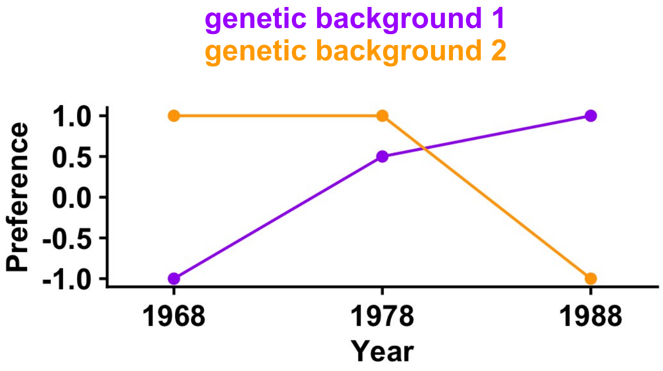

Supplementary Figure 4. Schematic overview of analyzing preference of natural occuring variant on a focal genetic background. The HA protein sequences of residues 156, 158, 159, 190, 193, 196 in naturally occurring H3N2 strains were extracted. In this simplified example here, three naturally occurring strains were used for demonstration. They were isolated from 1968, 1978, and 1988, respectively. The preference of each of these natural variants in different focal genetic backgrounds was plotted, with the year of strain isolation on the x-axis. There are only two focal genetic backgrounds in this example here. Our actual analysis in Fig. 3a included 45,218 human H3N2 strains that were isolated from 1968 to 2018, and six focal genetic backgrounds (HK68, Bk79, Bei89, Mos99, Bris07, or NDako16). Instead of plotting the preference of individual natural strains, the mean preference of all natural strains from a single year of isolation is plotted in Fig. 3a.

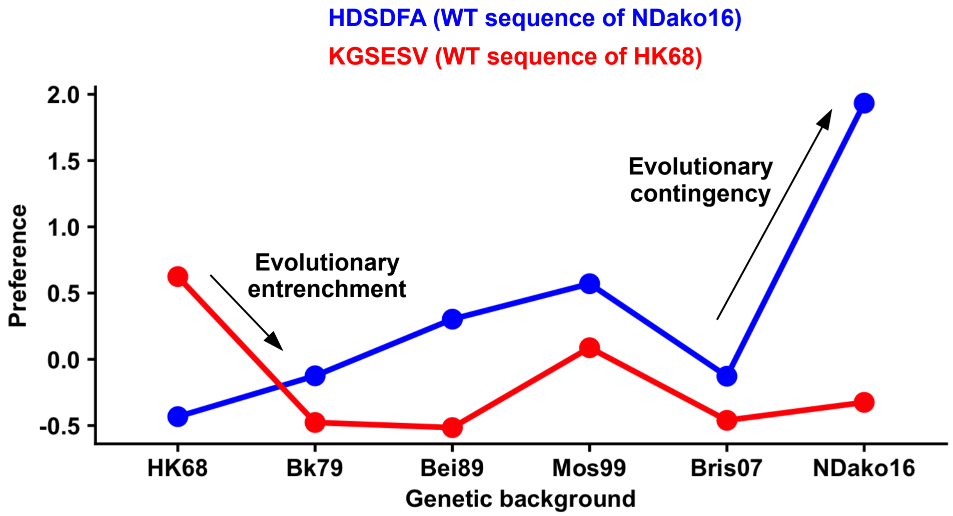

Supplementary Figure 5. Examples of evolutionary entrenchment and contingency. The preferences of HDSDFA (blue) and KGSESV (red) in different genetic backgrounds are shown. HDSDFA is the WT amino-acid sequence of NDako16 at residues 156, 158, 159, 190, 193, and 196. KGSESV is the WT amino-acid sequence of HK68 at residues 156, 158, 159, 190, 193, and 196. Evolutionary entrenchment describes sequence variants that are previously fit and then become unfit over time, as exemplified by KGSESV. KGSESV was fit in HK68, but became unfit in Bk79. Evolutionary contingency describes sequence variants that are previously unfit and then become fit over time, as exemplified by HDSDFA. HDSDFA was unfit in Bris07, but became fit in NDako16.

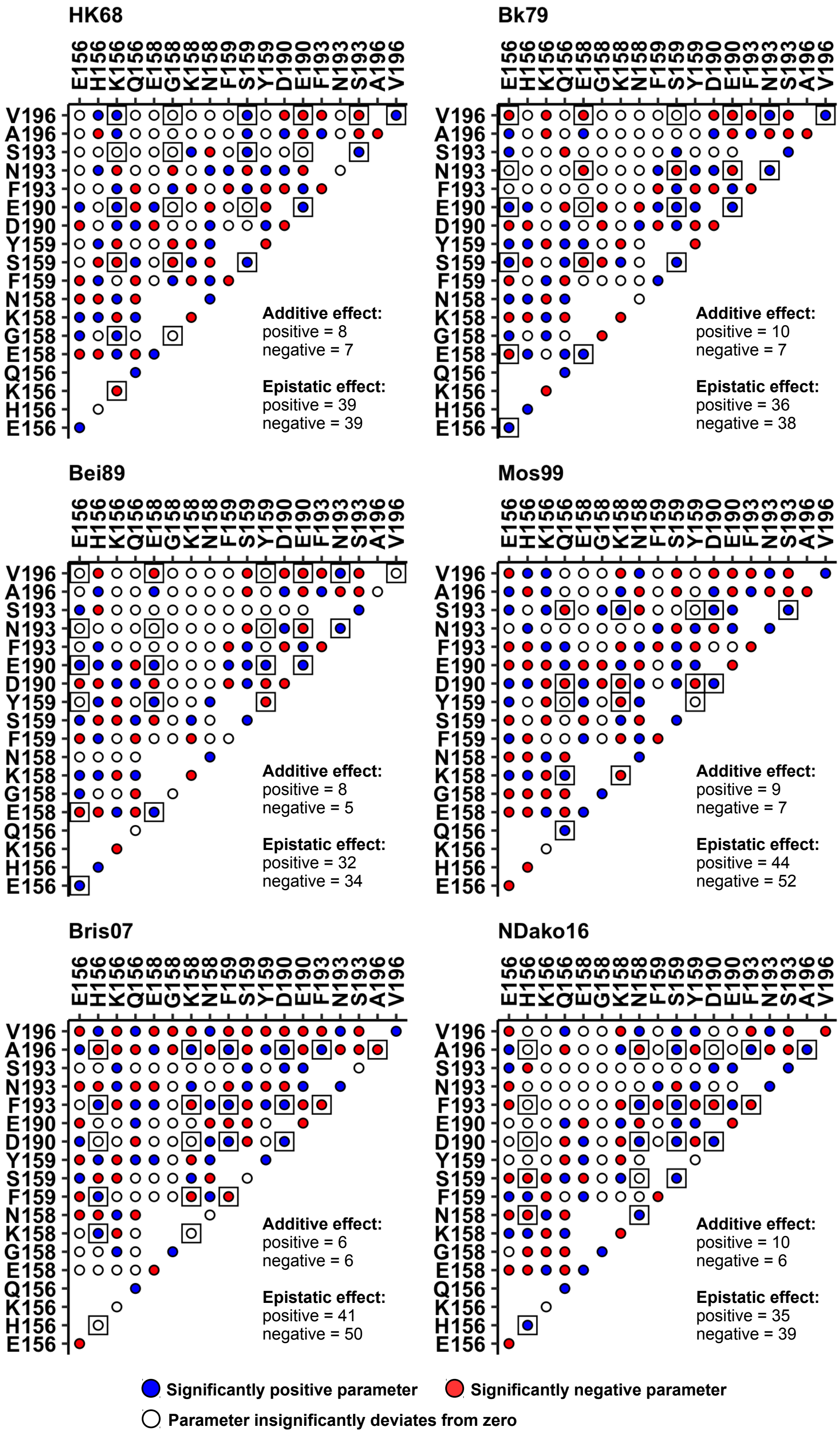

Supplementary Figure 6. Sign of parameters for additive fitness and pairwise epistatic effects. For each genetic background, the parameter for each additive fitness effect and pairwise epistatic effect at the residues (additive effect) or residue pairs (epistatic effect) of interest is computed. Parameters that are significantly positive at 95% confidence interval are shown in blue circles. Parameters that are significantly negative at 95% confidence interval is shown in red circles. Parameters that are neither significantly positive or negative at 95% confidence interval are shown in white circles. Parameters that contribute to the wild-type sequence for each genetic background are boxed. The number of positive and negative parameters are indicated at the bottom right of each panel.

a

2Fo-Fc map

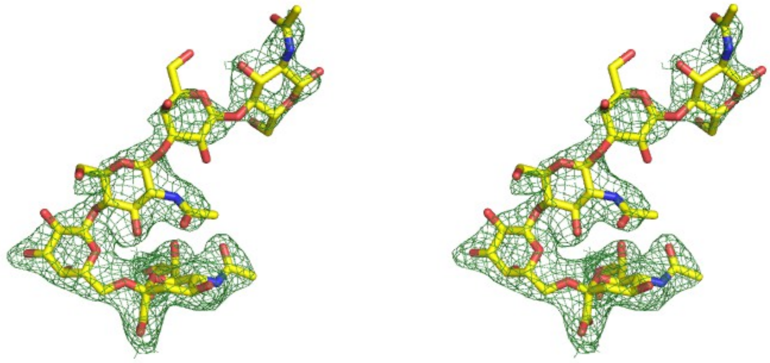

b

Omit (Fo-Fc) map

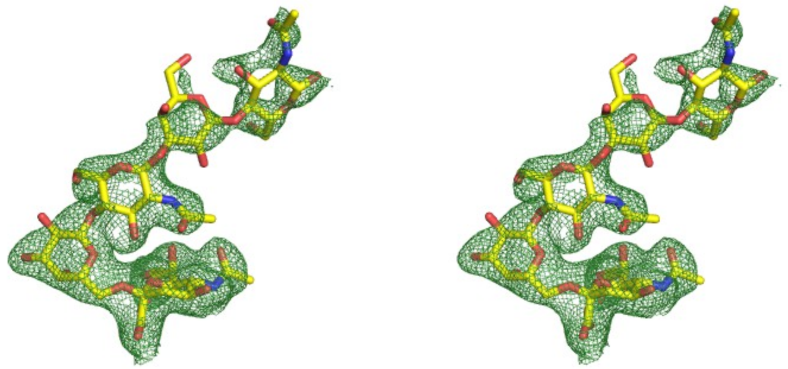

Supplementary Figure 7. Final 2Fo-Fc and composite omit (Fo-Fc) electron density maps of human glycan receptor analog 6′SLNLN. (a) Stereo representation of the final 2Fo-Fc electron density map for the human glycan receptor analog 6′SLNLN (yellow sticks) is represented in a green mesh and contoured at 0.8 σ. **(b)** Stereo representation of the composite omit (Fo-Fc) electron density map for the glycan receptor analogs is represented in a green mesh and contoured at 2.0 σ.

a

	Residues					
**Strain**	**135**	**137**	**145**	**222**	**225**	**226**
HK68	G	N	S	W	G	L
Bk79	G	Y	N	W	G	L
Bei89	E	Y	K	W	G	L
Mos99	T	S	N	W	G	I
Bris07	T	S	N	R	N	I
NDako16	T	S	S	R	D	I

b

Pairwise amino-acid sequence identities of the RBS base

**Bk79**	4				
**Bei89**	3	4			
**Mos99**	2	3	2		
**Bris07**	0	1	0	4	
**NDako16**	1	0	0	3	4
	**HK68**	**Bk79**	**Bei89**	**Mos99**	**Bris07**

c

Pairwise amino-acid sequence identities of the HA ectodomain

**Bk79**	460				
**Bei89**	446	476			
**Mos99**	438	454	467		
**Bris07**	429	447	454	466	
**NDako16**	424	441	446	453	476
	**HK68**	**Bk79**	**Bei89**	**Mos99**	**Bris07**

Supplementary Figure 8. Pairwise amino acid-sequence identities. (a) Amino-acid sequences of the RBS base, namely residues 135, 137, 145, 222, 225, and 226, are shown. **(b)** Pairwise amino-acid sequence identities of the RBS base are shown. **(c)** Pairwise amino-acid sequence identities of the entire HA ectodomain are shown.

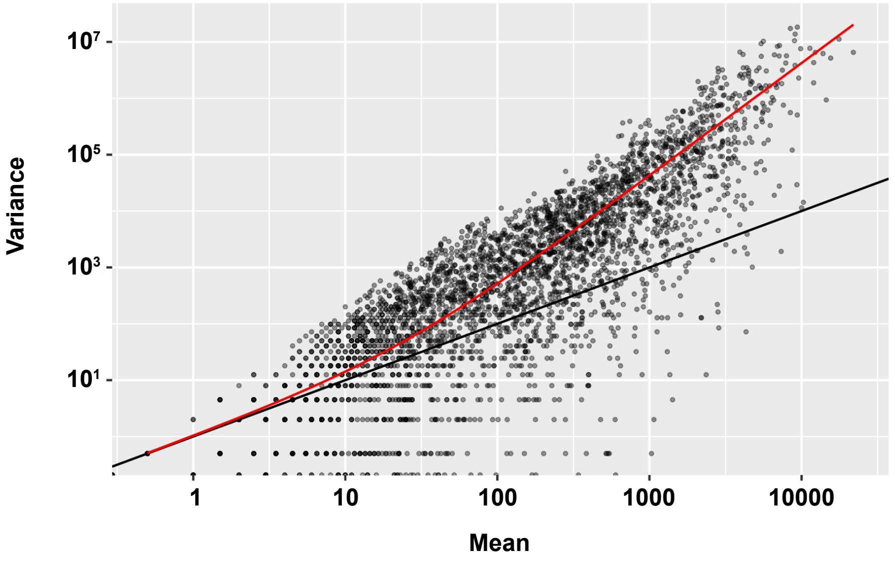

Supplementary Figure 9. Mean and variance of counts in two replicate measurements. Each point represents a sequence variant. The black line has slope of unity and represents the Poisson expectation, while the red line shows the relationship between mean and variance in the inferred negative binomial model.

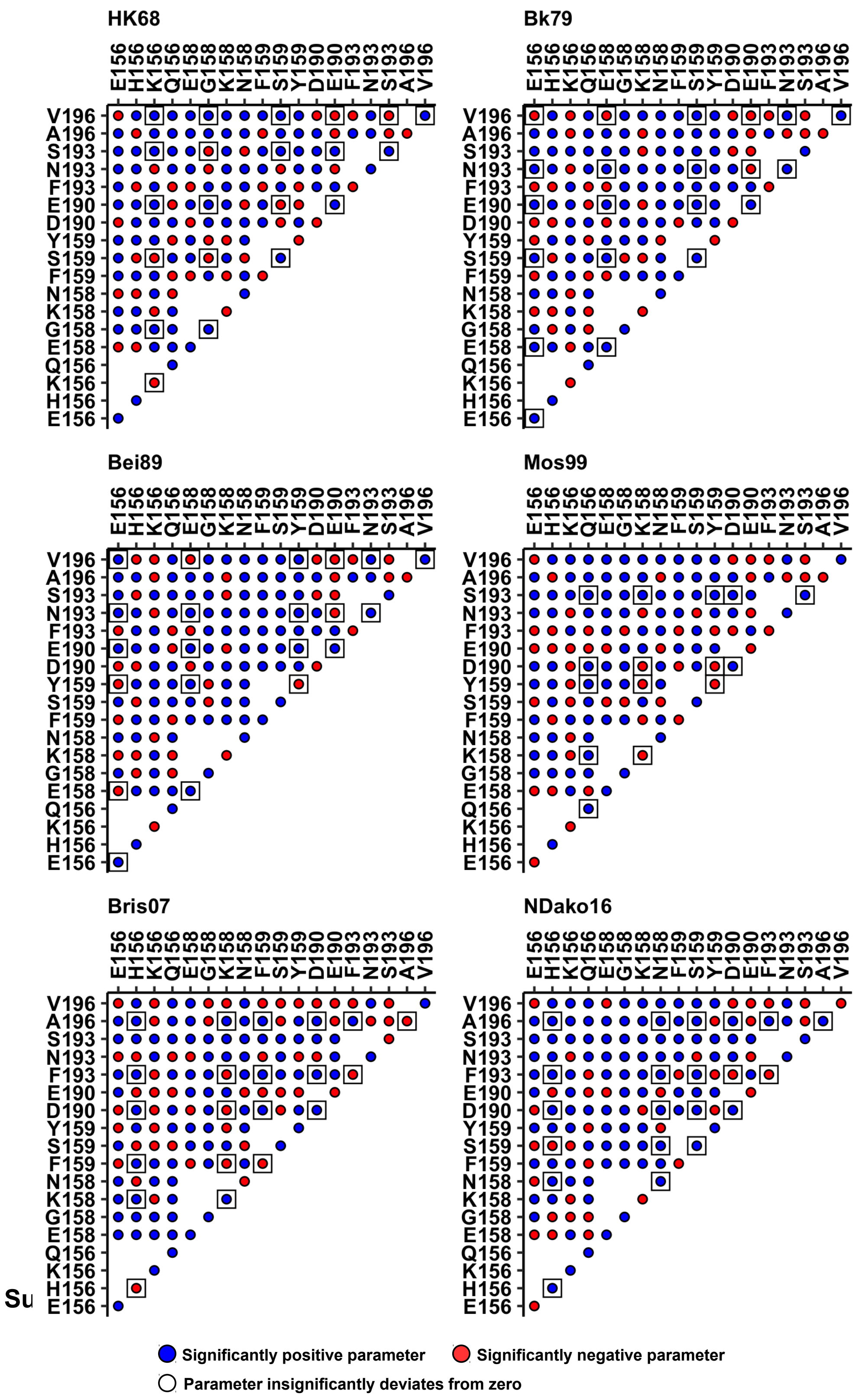

Supplementary Figure 10. Additive fitness and pairwise epistatic effects in a model without non-linearity. This analysis is the same as Supplementary Fig. 6, except that the additive fitness and pairwise epistatic effects were computed using a model without non-linearity.

Supplementary References

1. Chen VB*, et al.* MolProbity: all-atom structure validation for macromolecular crystallography. *Acta Crystallogr D Biol Crystallogr* **66**, 12-21 (2010).
