# Supplementary material for: Major antigenic site B of human influenza H3N2 viruses has an evolving local fitness landscape
Source: Nat Commun. 2020 Mar 6;11:1233. doi: 10.1038/s41467-020-15102-5 (PMC7060233; doi:10.1038/s41467-020-15102-5)
Supplement: Supplementary file 4 — Description of Additional Supplementary Files [file 41467_2020_15102_MOESM4_ESM.pdf]

Description of Additional Supplementary Files

File Name: Supplementary Data 1
Description: Protein sequences of the HA ectodomains from the six human influenza H3N2 strains of interest.

File Name: Supplementary Data 2
Description: The preference and unnormalized fitness of each of the 576 influenza virus variants in different genetic backgrounds.

File Name: Supplementary Data 3
Description: Full-length HA protein sequences from 45,218 human H3N2 strains that were analyzed in this study.
